# Lebrikizumab-induced psoriasis in a patient with atopic dermatitis

**DOI:** 10.1016/j.jdcr.2025.11.003

**Published:** 2025-11-08

**Authors:** Marley Cutrona, Alexandra K. Golant

**Affiliations:** Department of Dermatology, Icahn School of Medicine at Mount Sinai, New York, New York

**Keywords:** anti-IL-13-antibody, atopic dermatitis, lebrikizumab, paradoxical psoriasis, paradoxical reaction, psoriasis

## Introduction

While biologic therapies have revolutionized the management of chronic inflammatory skin diseases such as atopic dermatitis (AD) and psoriasis, these targeted treatments can sometimes trigger paradoxical cutaneous reactions.^1^^,^^2^ AD and psoriasis have historically been considered distinct entities, with AD characterized predominantly by T helper type 2 (Th2) mediated immune responses, and psoriasis by T helper type 1 (Th1) and T helper type 17 (Th17) responses.^3^ However, clinical overlap and treatment-induced phenotype shifts have increasingly been recognized.^1^^,^^3^

Targeted biologic therapies can disrupt the Th1/Th17-Th2 immune balance, leading to paradoxical skin reactions.^1^ In psoriasis, tumor necrosis factor-α inhibitors, interleukin (IL)-17 inhibitors, and IL-12/IL-23 inhibitors have been associated with eczematous eruptions.^1^ Conversely, in AD, the emergence of psoriasis or psoriasiform eruptions has been reported, most notably with dupilumab, an IL-4/IL-13 inhibitor, and more recently with tralokinumab, a selective IL-13 inhibitor.^2^^,^^4^^,^^5^

Lebrikizumab, another monoclonal antibody that selectively inhibits IL-13, was recently approved for the treatment of moderate to severe AD. Lebrikizumab demonstrated a favorable tolerability and safety profile in clinical trials, with conjunctivitis, infection, and injection-site reactions among the most common adverse events.^6^ Notably, psoriasis or psoriasiform eruptions after drug initiation have not been documented to date. Here, we present a case of paradoxical psoriasis following treatment with lebrikizumab in a patient with AD, providing new insight into the role that selective IL-13 inhibition may play in this paradoxical skin reaction.

## Case report

**Fig 1:**
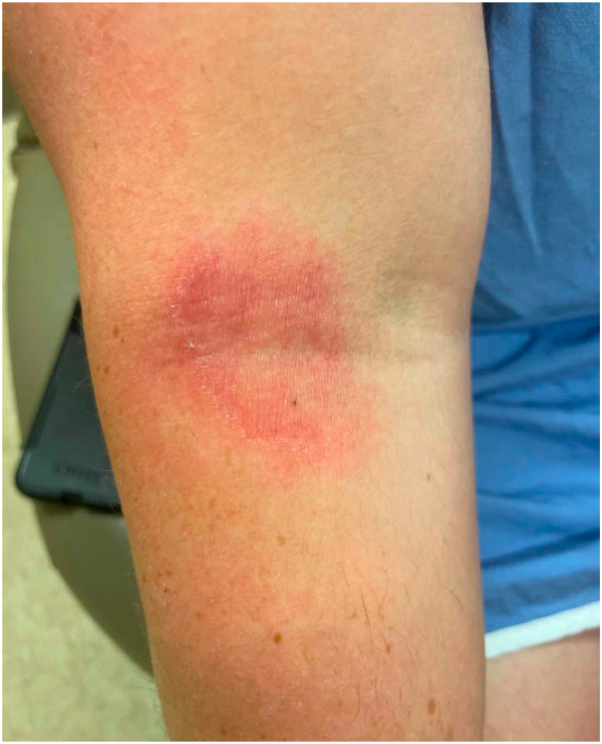
Clinical presentation of AD prior to the initiation of lebrikizumab therapy with an erythematous, scaly patch on the right antecubital fossa. *AD*, Atopic dermatitis.

**Fig 2:**
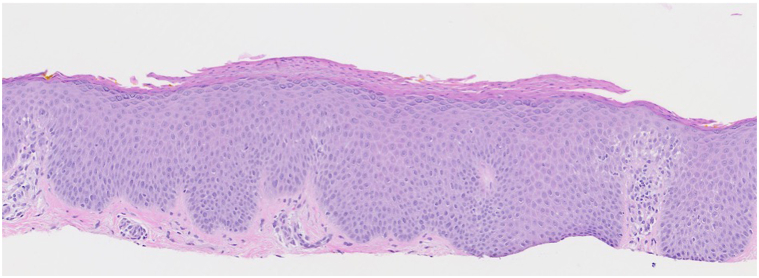
Hematoxylin and eosin (H&E)-stained section from skin biopsy of the right upper arm (10×) showing chronic spongiotic dermatitis.

A 29-year-old man with AD presented to our dermatology clinic for evaluation. He reported a personal history of seasonal allergies and childhood asthma but had no personal or family history of psoriasis. His previous treatments for AD included various steroidal and nonsteroidal topicals as well as systemic therapy with dupilumab and tralokinumab, both resulting in suboptimal disease control. Upon initial evaluation, the patient had significant pruritus as well as scattered eczematous skin lesions involving the face, neck, and extremities (Fig 1). A skin biopsy was performed, which showed chronic spongiotic dermatitis, and the decision was made to initiate treatment with upadacitinib 15 mg daily (Fig 2). Despite initial improvement with upadacitinib, therapy was discontinued due to waning efficacy as well as hyperlipidemia. The patient was subsequently transitioned to abrocitinib 100 mg daily.

After 3 months of abrocitinib, incomplete skin clearance prompted the addition of lebrikizumab to the treatment regimen. Lebrikizumab was administered at standard dosing (500 mg loading dose at week 0 and week 2, followed by 250 mg biweekly), leading to rapid improvement of his skin lesions and itch. The patient was successfully able to discontinue abrocitinib after 2 months of this combination therapy and was subsequently able to transition to lebrikizumab maintenance dosing (250 mg every 4 weeks) following 4 months of treatment, all while maintaining skin and itch clearance.

**Fig 3:**
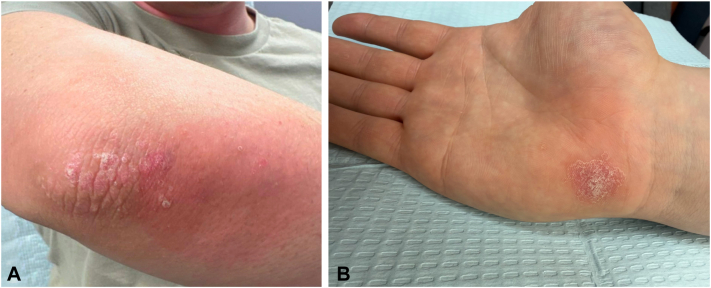
Clinical presentation of lebrikizumab-induced psoriasis with erythematous, scaly plaques on the **(A)** right elbow and **(B)** right palm.

**Fig 4:**
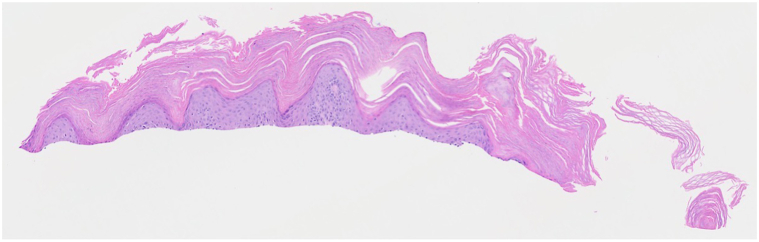
Hematoxylin and eosin (H&E)-stained section from skin biopsy of the right elbow (20×) showing features consistent with psoriasis.

At routine follow-up 7 months after starting lebrikizumab (corresponding to 5 months of monotherapy treatment), the patient reported that he had developed a new rash on his elbows. The exam revealed well-defined, erythematous plaques with thick scale localized to the extensor surfaces of the elbows bilaterally (Fig 3, *A*). A skin biopsy was performed, which showed confluent parakeratosis with collections of neutrophils, a thin granular layer, regular epidermal hyperplasia with elongated rete ridges, thin suprapapillary plates, and dilated blood vessels in the papillary dermis, confirming a diagnosis of psoriasis (Fig 4). The patient opted to continue lebrikizumab and add nonsteroidal topicals to spot treat this area. He was seen for follow-up 3 months later, where he reported new-onset plaques in the scalp and on the palm (Fig 3, *B*).

## Discussion

To our knowledge, lebrikizumab-induced psoriasis has not previously been reported, and clinical trials of lebrikizumab do not include psoriasis or psoriasiform eruptions as an adverse event.^6^ In contrast, in a large cohort study assessing AD patients treated with dupilumab, the 3-year cumulative incidence of psoriasis was 2.86% compared with 1.79% in the control group (*P* < .001).^4^ Published safety data now includes psoriasis and psoriatic arthritis as recognized adverse effects of dupilumab therapy. Notably, these adverse events were not observed in the pivotal dupilumab clinical trials for AD and have largely emerged through postmarketing surveillance and real-world safety studies.^4^ Given the more recent approval of lebrikizumab, real-world safety data remains limited, and its adverse event profile may not yet be fully characterized.

Paradoxical psoriasis following treatment with IL-4/IL-13 inhibitors like dupilumab, but historically not with selective IL-13 inhibitors, has led to the hypothesis that IL-4 blockade is the key driver of this phenomenon. Transcriptomic analyses of dupilumab-induced psoriatic eruptions revealed upregulation of Th17/IL-23 pathways and a reduction in Th2 gene expression.^7^ IL-4 exerts a suppressive effect on the Th17 pathway; therefore, IL-4 inhibition may shift immune responses toward Th17-mediated inflammation, a central pathway in psoriasis pathogenesis.^5^^,^^8^ This mechanism was further supported by Quattrini et al, who reported 2 cases of dupilumab-induced psoriasis successfully treated with tralokinumab, suggesting that IL-4 inhibition alone was responsible for this paradoxical reaction.^9^

However, emerging evidence challenges the notion that IL-4 blockade is solely responsible. Psoriasis has previously been described in an AD patient following treatment with tralokinumab and now in our patient following treatment with lebrikizumab, with both drugs sharing a similar mechanism of action by selectively inhibiting IL-13. This suggests that IL-13, in addition to IL-4, plays an important role in maintaining immune balance among the Th1/Th17 and Th2 pathways. Supporting this, genetic studies have demonstrated that genetically mimicked IL-13 inhibition is associated with an increased risk of psoriasis and psoriatic disease.^10^

This case of lebrikizumab-induced psoriasis in a patient with AD supports the concept that inhibition of IL-13, like IL-4, may play a role in shifting the immune response toward the Th17 axis, precipitating psoriatic disease. This represents the first reported case of psoriasis associated with lebrikizumab in a patient with AD and adds to the growing body of evidence implicating IL-13 inhibition as a potential contributor to psoriatic disease pathogenesis.

## Conflicts of interest

Dr Golant declares personal fees from AbbVie, Amgen, Apogee Therapeutics, Arcutis, Boehringer Ingelheim, Bristol Myers Squibb, Dermavant, Galderma, Incyte, Janssen, LEO Pharma, Lilly, Pfizer, Regeneron, Sanofi, and Takeda Pharmaceuticals. Dr Cutrona has no conflicts of interest to declare.
